# Surgical Approaches and Their Outcomes in the Treatment of Cubital Tunnel Syndrome

**DOI:** 10.3389/fsurg.2018.00048

**Published:** 2018-07-26

**Authors:** Adam Carlton, Syed I. Khalid

**Affiliations:** ^1^Chicago Medical School, Rosalind Franklin University, North Chicago, IL, United States; ^2^Department of General Surgery, Rush University Medical Center, Chicago, IL, United States

**Keywords:** cubital tunnel, ulnar nerve, ulnar neuropathy, peripheral nerve, outcomes

## Abstract

**Purpose:** This review was undertaken in order to provide an updated summary of the current literature on outcomes for various surgical treatments for cubital tunnel syndrome.

**Methods:** Studies reporting outcomes for surgical treatment of cubital tunnel syndrome were collected through the PubMed database. Study structure, number of participants/procedures, mean follow-up times, scoring scales, and outcomes were collected according to the type of surgery: open decompression, endoscopic decompression, minimal incision, subcutaneous transposition, intramuscular transposition, and submuscular transposition.

**Results:** Our findings indicate varying but comparable levels of success among all surgical techniques reviewed. Many different scoring scales were utilized, limiting direct quantitative comparison between most studies.

**Discussion:** While some studies directly compared two or more techniques, there was rarely a statistically significant difference between groups. In comparisons that did reach statistically significant differences, there were others yet that found no difference in comparing the same techniques.

**Conclusions:** None of the techniques in this review has demonstrated universal superiority above all others, but all appear to be effective in the treatment of cubital tunnel syndrome. The only consensus seems to be that transposition is preferred where the ulnar nerve tends to subluxate either on preoperative or intraoperative examination.

## Introduction

Cubital tunnel syndrome is the second most common cause of peripheral nerve entrapment in the upper limb after carpal tunnel syndrome (1). Sensory and motor symptoms are most common in this disorder and while pain is not a prototypical feature, it may still be present. (2) Sensory symptoms often begin as paresthesia or dysesthesia in the corresponding dermatome through the medial aspect of the forearm and into the fourth and fifth fingers, over time progressing to full anesthesia in the most severe cases. Motor symptoms often present initially with hand weakness or cramping, with subsequent ulnar clawing due to the weakness of the lumbrical muscles. Surgical intervention for this condition was described as early as 1816 by Henry Earle which sectioned the nerve above the elbow, curing the patient's pain but leaving her with claw-like paralysis of the fifth digit (3).

Modern surgical treatment for idiopathic ulnar neuropathy of the elbow has evolved through several phases, the two most prevalent techniques in use today being *in situ* decompression and transposition of the nerve anterior to the medial epicondyle, with several common permutations of each. Unless subluxation of the nerve is noted on preoperative or intraoperative examination, in which case anterior transposition is generally preferred, (4, 5) the choice of technique is largely left to surgeon preference with no clearly superior procedure having been identified to date. Medial epicondylectomy is generally only indicated when structural abnormalities of the anatomy are present, often due to either trauma or degenerative changes, and may be performed in combination with other techniques. Evaluation of outcomes in the context of the pros and cons of each technique may help guide future innovation in the treatment of this condition.

## Methods

Many different scoring scales are used across these studies, however most studies group outcomes into Excellent, Good, Fair, Satisfactory, and Poor. In order to approximate comparisons across scales, combined good and excellent percentages (CGE) were calculated where applicable. Results cited in the text have their score scale denoted by the corresponding abbreviation; papers that did not state the scale used are denoted (U).

### Bishop score (B)

Originally described by Kleinman and Bishop (6) the modified version as demonstrated by Schmidt et al. (7) is frequently used in more recent studies in which the patient evaluates their symptoms and functional abilities and then classifies them as Excellent, Good, Fair, or Poor outcomes based on the total score (Table 1A).

### Lsu grade (L)

Described in detail in Kline and Hudson's Nerve Injuries (2008) (8) and Kim et al. (9) describes sensory and motor findings on a 0–5 scale each, with an overall grading for ulnar nerve entrapments combining the two (Table 1B).

### Wilson and krout (WK)

Described by Wilson and Krout (10) more recent studies use a modified version that includes a poor outcome to describe no improvement (11, 12) which were not a part of the original scale which only used Excellent, Good, and Fair. (Table 1C).

### Mcgowan improvement (M)

The McGowan scale was originally described in 1950 as a simple grades 1–3 classification for the severity of disease based on sensory and motor examination findings (13) (Table 1D).

### Gabel amadio (GA)

The Gabel Amadio scale combines pain as well as sensory and motor exam findings on a scale from 0 to 3, the results are grouped into Excellent, Good, Fair, and Poor outcomes based upon score improvements (14) (Table 1E).

### Macdermid (McD)

A patient reported symptom, pain, and functional ability scoring scale, also known as the Patient Rated Ulnar Nerve Examination (PRUNE), the total score is calculated from 0 to 100 after summing each of the 20 items then dividing by two, with 0 being no symptoms or difficulty performing activities and 100 being worst possible symptoms or unable to do any functional activity (15) (Table 1F).

### Messina classification (Me)

Described by Messina and Messina grouping patients into Excellent, Good, Fair, and Poor groups based on the postoperative improvement of motor and sensory symptoms (16) (Table 1G).

### Subjective assessment (SA), patient satisfaction (PS), unstated (U)

Many studies utilize some combination of patient and/or clinician subjective rating or do not state a specific standardized scoring scale used.

## *in situ* release

### Open decompression

The first description of this technique is debated. Emile-Paul Fèvre, in 1878, described liberation and elongation of the nerve, (17) but Farquhar Buzzard in 1922 saw “fairly good results from merely dividing the connective tissue strands by means of longitudinal incisions” ultimately preferred an anterior transposition technique to ensure complete relaxation of the nerve (18). While the modern application of this technique varies to some degree between surgeons, it primarily uses a single longitudinal incision over the cubital tunnel providing visual exposure several centimeters both proximally and distally to allow for adequate decompression under direct visualization along the entire course of the elbow. As the ulnar nerve can commonly be compressed in several places as far proximally as the arcade of Struthers, down through the cubital tunnel and to the distal Osborne's ligament, (19) the open approach allows direct appreciation of compressive sites allowing the surgeon to ensure freedom of movement along the entire course of the nerve. However, the most frequent site of compression is still a point of contention and no uniform consensus exists as of yet (1).

The studies with the poorest outcomes as defined by combined good and excellent outcome percentages (CGE) were Bartels et al. with 65.3% (*n* = 75, U) and Sousa et al. 67.2% (*n* = 64, WK) (20, 21). Those with the best CGE outcomes, both limited by notably small sample sizes, were Cho et al. and Keiner et al. with 100% (*n* = 10, B) and 94.1% (*n* = 17, U) (22, 23). All other studies ranged from 78 to 91% CGE (Table 2A).

The largest sample size of this technique studied to date was Nathan et al., which was a retrospective study of 102 procedures in 74 patients. This study is limited in that the grouping of patients into excellent, good, fair, or poor outcomes relied on self-reported percentages of numbness and tingling resolved by surgery, but yielded a CGE of 82% (24).

Complication rates, while not uniformly reported, are generally low with this technique. Most frequently incisional tenderness,(25, 26) as well as numbness in the distribution of the median antebrachial cutaneous nerve (MACN), (26) followed by the less common superficial infections and wound dehiscence. Incisional length varies widely, but often as long as 8–10 cm (24, 26) or more which poses a substantial threat of injury to the MACN, as well as increased postoperative pain and healing time which are established consequences of open surgery and large incisions.

### Endoscopic

Endoscopic assistance for cubital tunnel surgery was first described in 1995 by Tsai et al. utilizing a single smaller 2–3 cm incision directly between the medial epicondyle and olecranon with a 5 mm diameter glass retractor tube and a meniscus knife to release the overlying connective tissue (27). This permits endoscopic visualization of the nerve along its entire course and release of tissues overlying the nerve as much as 10 cm in both proximal and distal directions from the insertion site.

Use of the endoscopic technique has been much more thoroughly studied compared with the open approach, with 13 single technique studies identified. The largest single technique study of the endoscopic approach was done prospectively in 172 procedures in 148 patients by (28) demonstrating remarkably good results with 96% CGE (B), with only a 4% (*n* = 7) complication rate, 4 wound dehiscences, 2 cases of cellulitis, and 1 self-limiting postoperative hematoma (Table 2B).

In 1999, Tsai et al. published a series of 85 procedures in 76 patients demonstrating 87% (B) CGE (29). The poorest outcomes of this technique were documented by Stadie et al. and Mirza et al. with 70% (*n* = 32, M) and 69.6% (*n* = 92, GA) CGE respectively 30, 31. In 2010, Flores documented a CGE of 100% (*n* = 13, B) (32). The remainder of studies documented CGE ranging from 84.9 to 93.5%.

Three studies comparing endoscopic and open techniques were found. In 2009, Watts and Bain demonstrated a greater although not statistically significant different CGE (*p* = 0.229) between endoscopic (79%, *n* = 19, PS) and open (60%, *n* = 15, PS) groups, but did find a significantly lower rate of complications in the endoscopic group (*p* = 0.044) (33). In 2013, Dutzmann et al. found significantly better short-term outcomes in the endoscopic group, but long-term outcomes were equivalent (26). In 2015, a randomized double-blind study by Schmidt et al. found no significant difference in CGE outcomes between open (81.5%, *n* = 27, B) and endoscopic (82.8%, *n* = 29, B) groups (*p* = 0.47), however there was a higher incidence of hematoma with the endoscopic technique (*p* = 0.05) (7).

There does not appear to be concrete evidence for the superiority of the endoscopic technique compared with an open approach in terms of long-term outcomes, and conflicting evidence regarding the incidence of complications between the two. The main advantage of this minimally invasive technique is achieving a comparable outcome to the open technique, with the reduced trauma to surrounding tissues due to the much smaller incision size required, but the equipment needed adds expense as well as the need for more training leading to higher overall costs.

A counter-point made by Cobb et al. to the added expense of the endoscopic method is the economic benefit of earlier return to work, decreased surgical times and resultant decreased facility costs, which they estimated the societal benefit over anterior transposition to be “$522,565,775 for the year 2016” based on a projected total of 73,673 cases performed nationwide, but this number does not have the costs of endoscopic equipment and training subtracted from it which they state are difficult to quantify (28).

### Minimal incision

In 2002, Taniguchi et al. described a simple decompression procedure performed without the use of endoscopic visualization, utilizing a single 1.5–2.5 cm incision demonstrating a CGE of 77.8% (M) in 19 procedures, with only one complication of a superficial hematoma and no reports of infection, MACN injury, or painful scarring (34). This technique uses a comparable or slightly smaller incision than the endoscopic technique while taking advantage of the freely mobile skin around the elbow joint to achieve decompression over an 8–10 cm total distance, using scissors as the primary dissection tool.

A study of 66 patients in 2010 by Jeon et al. with 81% of patients with preoperative McGowan stage 1 and 2 having satisfactory outcomes (Me) with a 3% postoperative complication rate of 2 hematomas (35). Karthik et al. demonstrated a CGE of 80% (*n* = 46, B) (36) and Lan et al. had a procedural satisfaction rate of 88% with 70% showing symptomatic improvement (37) (Table 2C).

In 2007, Cho et al. compared 5 minimal incision procedures with 10 open decompressions finding 100% CGE (B) in both groups with no complications (22). Subsequently in 2013, Bolster et al. found no statistically significant difference (*p* = 0.628) in CGE between the minimal incision approach (93%, *n* = 22, B) and the endoscopic technique (91%, *n* = 20, B), with the only complication reported being one case of wound infection in the endoscopic group (38).

While there is insufficient data to draw direct conclusions comparing outcomes between the two minimally invasive techniques, these studies do suggest the use of the endoscope may not, in fact, be necessary in order to achieve a comparable outcome. Despite the overall length of decompression being shorter than with open and endoscopic techniques, a review by Assmus et al. noted the most common sites of compression to be from the retrocondylar groove to Osborne's ligament which is the segment best appreciated with this technique (39). This technique strives to achieve the benefits of the minimally invasive endoscopic technique without the associated added equipment and costs.

## Anterior transposition

### Subcutaneous

In 1898 Benjamin Curtis was the first to describe anterior transposition of the ulnar nerve, placing it into the subcutaneous tissue (40). As described by Nabhan et al. the bulk of the exposure is done similar to that of the open approach, but additional subcutaneous dissection is performed anterior to the medial epicondyle, forming a bed in which the ulnar nerve may sit. A sling is then created from the underlying muscle fascia and attached to the dermis to retain the nerve in its newly transposed position (41).

Only two single technique studies have evaluated subcutaneous transposition. (12) retrospectively analyzed 33 cases finding 94% CGE (WK) with no complications. In 2015, Lima et al. demonstrated 77.7% CGE (*n* = 36, B) with complications of scar pain (*n* = 5), paresthesia (*n* = 4), and early superficial infection (*n* = 1) (Table 2D).

This technique has been studied more extensively in comparison with other techniques, generally showing no significant difference between *in situ* decompression and subcutaneous transposition outcomes (20, 21, 41–43).

Mitsionis et al. found that subcutaneous transposition had inferior outcomes to *in situ* decompression (*p* < 0.05) (44). Vanaclocha et al. demonstrated better motor and sensory outcomes in the *in situ* decompression group than the subcutaneous transposition group despite there being no significant difference between the groups in both pre- and postoperative motor and sensory nerve conduction velocity measurements (45).

A recent meta-analysis by Chen et al. concluded that outcomes were equivalent between subcutaneous transposition and *in situ* decompression (*p* = 0.891), however, subcutaneous transposition had a significantly higher complication rate (*p* = 0.05) (46)

Overall, there is no evidence to suggest subcutaneous transposition is superior to *in situ* decompression, and that outcomes are likely comparable between the two techniques. Except in the case of a nerve subluxation on exam, which over time may cause chronic irritation which is relieved by transposition it may be preferable to perform *in situ* decompression as the *de facto* procedure in order to preserve the vascular supply which is disrupted by transposition, however many proponents of the procedure argue that the anastomoses between proximal and distal vascular supply to the nerve negates this point (47). The nerve is more exposed to potential trauma in its post-transposition location, with only the skin and small amount of subcutaneous tissue protecting it from external forces as compared to being protected by the bony structures of the elbow and several layers of overlying tissue in its native position.

### Intramuscular

In 1917, Rudolf Klauser described a variant of the anterior transposition placing the nerve into the plane between the pronator teres and flexor carpi ulnaris muscles (48). In 1989, Kleinman et al. retrospectively analyzed 52 procedures in 48 patients, finding a CGE of 87% (B). They noted that many detractors of the technique previously were concerned about scarring within the muscle bed or traction forces on the nerve, but these concerns have yet to be proven and no complications were noted in this study (6) (Table 2E).

Only one comparative study, Emamhadi et al. was found, finding intramuscular transposition (*n* = 40) to have better motor outcomes than subcutaneous transposition (*n* = 43), but equivalent pain and sensory outcomes between the two groups (49). It was posited by Kleinman that adequate release of the fibrous aponeurosis and intermuscular septum between the flexor and pronator muscles in addition to the creation of a 5 mm trough fashioned into the musculature allows for free movement of the ulnar nerve in a well-vascularized bed (6) providing a better environment for healing and protection than the subcutaneous location.

Intramuscular transposition may be a viable technique, but it is clearly non-dominant in today's practice and little is known about associated outcomes. Further studies are needed to assess its outcomes relative to the other subcutaneous and submuscular transposition techniques.

### Submuscular

Submuscular transposition was first described in 1942 by Sir James Learmonth (50). The technique, as described by Davis and Bulluss is initially analogous to *in situ* decompression, releasing the same tissues, and then after dissecting the pronator teres and necessary portions of the flexor carpi ulnaris from the medial epicondyle, buries the nerve underneath the pronator teres muscle with subsequent reattachment to the medial epicondyle (51). Some modified techniques specify a Z-lengthening of the flexor-pronator mass to decrease tension over the nerve in its new location (52).

Both Gervasio and Gambardella with a CGE of 87% (*n* = 18, B), (52) and Davis and Bulluss with 82.5% of patients (*n* = 40 procedures) improving at least one LSU grade, (51) have demonstrated good results with this technique, with only one complication of MACN distribution numbness between the two studies. (Table 2F).

Four studies have compared this technique against subcutaneous transposition, three demonstrating no significant difference in outcomes (43, 45, 53) and one, Jaddue et al. indicating significantly better outcomes with subcutaneous transposition (*p* = 0.035). (54) In comparing submuscular transposition with *in situ* decompression, six studies have found no significant difference in outcomes between the two techniques. (23, 25, 43, 45, 55, 56).

Bimmler et al. argued that “simple decompression of the ulnar nerve can be recommended in all patients without cubital (sub)luxation of the nerve, whereas people with a tendency of cubital (sub)luxation of the ulnar nerve should be treated by submuscular anterior transposition”(55).

The main advantage of this when compared with the other transposition techniques is the protection offered by the overlying muscle, but there are no studies that demonstrate any degree of superiority over any of the techniques discussed. Submuscular placement may be preferable when the patient has little subcutaneous tissue to protect the nerve but transposition is necessary due to subluxation of the nerve.

## *In situ* decompression vs. anterior transposition for ulnar nerve subluxation

While anterior transposition is widely accepted as the preferred method for treating cubital tunnel syndrome where ulnar nerve subluxation is present, there do not appear to be any studies specifically comparing *in situ* decompression against anterior transposition in this specific subset of patients. One study, Bimmler and Meyer which compared simple decompression against anterior transposition, stated that the specific group of patients with subluxation of the nerve experienced distinctly better results when treated with anterior transposition rather than simple decompression, but that overall there was no significant difference between the two groups (55). Beyond that study there does not appear to be any evidence supporting the widely held belief that transposition is superior for this subset of patients.

## Discussion

Despite the different scoring scales used and difficulty comparing studies directly, the bulk of single technique outcomes studies and multi-technique comparative studies demonstrate that all surgical techniques discussed are effective treatment methods for cubital tunnel syndrome, but fail to demonstrate any one technique to be uniformly superior to another, except in the case of ulnar nerve subluxation in which transposition is generally preferred. While there is little evidence to support the preferential use of transposition in the setting of subluxation, this does seem to be a logical solution based upon biomechanics which may explain its dominance.

The difference in value between functional and objectively measurable outcomes is uncertain. While measurable improvements in nerve conduction velocities suggest the success of an operation, one study found no correlation between functional and neurophysiological outcome in submuscular transposition (51). While some studies use improvements in nerve conduction velocities as an objective measure of surgical success, what matters most to patients is going to be the reduction of symptoms such as weakness, numbness, and pain, and the regain of normal function. For this reason, we suggest that future studies should use objective measures of function such as grip strength, two-point discrimination, and standardized objective score scales.

We suggest using the LSUMC classification system for ulnar nerve entrapments for clinicians to grade pre- and postoperative severity of motor and sensory symptoms as used by Biggs and Curtis (56). because of its improved granularity over the McGowan system, and the common modified Bishop Score scale used by Schmidt et al. (7). which provides a standard framework in which to assess patient satisfaction in terms of function and symptom improvement.

When considering the various techniques with roughly equal efficacy, many authors suggest defaulting to techniques that minimize incision size and degree of tissue dissection, operating time, post-operative complication rates. While the current trend in many historically open surgical procedures, such as the appendectomy and cholecystectomy, is to now use smaller incisions and the aid of endoscopic visualization, no comparative studies in this review demonstrated significantly better outcomes with use of the endoscope. Because of this, we question the purported benefits and resultant added expense of using endoscopes in treating cubital tunnel syndrome. In an era of ever rising healthcare costs, this represents one potential source of cost reduction.

Going forward it will be of great importance for studies to focus on the economic side of these procedures in addition to clinical outcomes as we surmise there may genuinely not be one technique that provides superior clinical outcomes. Cost analyses, return to work time as it relates to societal economic cost, post-operative complication rates (especially those leading to readmission, disability, or delayed recovery), and surgical time may come to be the benchmark by which these techniques are stratified.

## Limitations

This review has a number of limitations in comparing the different techniques. The most glaring is the wide variety of scales, both objective and subjective, used to determine the surgical outcome, preventing directly quantifiable comparisons between most studies. Length of symptoms preoperatively and mean follow-up times differ substantially across studies, each of which may have considerable impact on outcomes. There is also variability in techniques and procedural familiarity between surgeons that may lead to biased results, especially if one surgeon is comparing a technique they rarely perform against one they use frequently.

## Conclusions

Several techniques have evolved from the original accounts of liberation and elongation and anterior transposition. The primary driving force in most surgical fields is to use less invasive techniques that are known to lead to shorter healing times, less pain, decreased operative times, and decreased rates of infection which have guided the evolution of surgical treatment of cubital tunnel syndrome.

The literature suggests that all of these techniques are generally effective, and that surgeon preference is the predominant deciding factor in most cases, but some are more invasive than others which is an important consideration going forward. Perhaps the only consensus to date is that transposition should be performed when subluxation of the nerve is present. Outside of that constraint, with all other factors being equal, a minimally invasive and cost-effective approach to surgery should be a primary goal.

Despite the apparent effectiveness of all techniques, none have been established to be uniformly superior to the rest. There is a stark need for larger studies using common standardized objective scoring scales in order to better understand the advantages and disadvantages of each technique and be able to compare results from one study to the next.

## Author contributions

AC and SK both contributed to the gathering of data, writing, and final editing and preparation of the manuscript.

### Conflict of interest statement

The authors declare that the research was conducted in the absence of any commercial or financial relationships that could be construed as a potential conflict of interest. The reviewer WL and handling Editor declared their shared affiliation.

## Abbreviations

CGE: Combined Good and Excellent Percentages
MACN: Median Antebrachial Cutaneous Nerve
PRUNE: Patient Rated Ulnar Nerve Examination
B: Bishop Score
LSU: Louisiana State University
M: McGowan
GA: Gabel Amadio
McD: MacDermid
Me: Messina
SA: Subjective Assessment
PS: Patient Satisfaction
U: Unstated.
